# Promoter Hypermethylation of ARID1A Gene Is Responsible for Its Low mRNA Expression in Many Invasive Breast Cancers

**DOI:** 10.1371/journal.pone.0053931

**Published:** 2013-01-21

**Authors:** Xianyu Zhang, Qian Sun, Ming Shan, Ming Niu, Tong Liu, Bingshu Xia, Xiaoshuan Liang, Wei Wei, Shanshan Sun, Youxue Zhang, Xiaolong Sean Liu, Qingbin Song, Yanmei Yang, Yuyan Ma, Yang Liu, Long Yang, Yanlv Ren, Guoqiang Zhang, Da Pang

**Affiliations:** 1 Department of Breast Surgery, The Third Affiliated Hospital of Harbin Medical University, Harbin, Heilongjiang, China; 2 Department of Pathology, The Third Affiliated Hospital of Harbin Medical University, Harbin, Heilongjiang, China; 3 Department of Medicine, Mount Sinai School of Medicine, New York, United States of America; 4 Key Laboratory, Heilongjiang Institute for Cancer Research, Harbin, Heilongjiang, China; 5 ICFC Custom Service, Life Technologies Corporation, Beijing, China; Vanderbilt University Medical Center, United States of America

## Abstract

ARID1A (AT-rich interactive domain 1A) has recently been identified as a tumor suppressor gene. Its mRNA expression is significantly low in many breast cancers; this is often associated with more aggressive phenotypes. However, the underlying molecular mechanism for its low expression has not been fully understood. This study was undertaken to evaluate the contribution of gene copy number variation, mutations, promoter methylation and histone modification to ARID1A’s low expression. 38 pairs of breast invasive ductal carcinomas and their normal breast tissue counterparts from the same patients were randomly selected for gene expression and copy number variation detection. Promoter methylation and histone modification levels were evaluated by MeDIP-qPCR and ChIP-qPCR, respectively. PCR product Sanger sequencing was carried out to detect the exon mutation rate. Twenty-two out of 38 invasive ductal carcinomas in the study (57.9%) revealed ARID1A mRNA low expression by realtime RT-PCR. The relative promoter methylation level was, significantly higher in ARID1A mRNA low expression group compared with its high expression group (*p*<0.001). In the low expression group, nineteen out of 22 invasive ductal carcinomas (86.4%) exhibited ARID1A promoter hypermthylation. In addition, the promoter hypermethylation was accompanied with repressive histone modification (H3K27Me3). Although five out of 38 invasive ductal carcinomas (13.2%) exhibited loss of ARID1A gene copy number by realtime PCR and nine exon novel mutations are seen from eight out of 33 invasive ductal carcinomas (24.2%), there was no statistically significant difference in both ARID1A mRNA low and high expression groups (*p* = 0.25,and *p* = 0.68, respectively). We demonstrate that promoter hypermethylation was the main culprit for ARID1A mRNA low expression in invasive ductal carcinomas. The influence of mutation and copy number variation on the expression were statistically insignificant at mRNA level, and were, therefore, not considered the main causes for ARID1A mRNA low expression in invasive breast cancer.

## Introduction

ARID1A (synonyms: p270, BAF250a, hOSA1, SMARCF1) gene is located on 1p36.11. Its gene product, BAF250a, is a member of the ARID (A-T-rich interaction domain) family of DNA-binding proteins [1], [2] and a subunit of human SWI/SNF-related complexes, which use the energy generated by an integral ATPase subunit to remodel chromatin.

BAF250a as the ARID1A gene product is, however, frequently deficient in tumor tissue samples and breast cancer cell lines [3], and is thus implicated in the tumor suppressor function of the complexes [4]. ARID1A is known as an essential gene for FAS-mediated apoptosis [5]. The interaction between BAF250a and promoter is correlated with repression of the cell-cycle-specific genes [6], and the BAF250a-depleted cells induced by siRNA fail to undergo normal cell cycle arrest [7]. The role of BAF250a/ARID1A in cell-cycle repression implies that it contributes significantly to the tumor suppression activities of SWI/SNF complexes.

Molecular mechanisms for low ARID1A mRNA and protein expression appear to be different among various types of malignant tumors. For example, ARID1A gene mutations result in the low protein expression in ovarian clear cell carcinoma (with mutation rate ∼50%) [8], [9]; however, in pancreatic cancer, frequent copy number loss (47%) is the main cause [10].

We have previously reported that ARID1A mRNA and protein expression is decreased in 56% of breast cancer tissues [11]. Similar finding is reported (64%) by Mamo [12]. Additionally, ARID1A gene mutations and copy number loss have been reported in some breast cancers. One previous study indicates ARID1A gene mutation rate is about 4% in breast cancers, but its copy number loss occurs in 13% in breast cancers [12] reported by the same group and 35% reported by another group [13], respectively. It is unclear that whether or not the ARID1A gene mutations and/or its copy number loss are responsible for the ARID1A gene low mRNA expression. It is also unclear that whether or not hypermethylation contributes to the ARID1A low mRNA expression. We carried out this study in order to evaluate the prevalence of ARID1A gene mutations, and copy number loss in breast cancers, and its relationship with ARID1A gene mRNA expression; in addition, we successfully demonstrate that promoter hypermethylation in ARID1A gene is strongly correlated with ARID1A gene low mRNA expression.

## Materials and Methods

### Samples

38 pairs of fresh frozen breast invasive ductal carcinomas and corresponding normal breast samples used in the current study were obtained from the Heilongjiang Breast Tumor Biobank, which operates with the approval of the Faculty of Harbin Medical University, Research Ethics Board. All patients signed the informed consent form (detailed clinical information can be found in Table S1). All specimens were immediately frozen in liquid nitrogen and kept at −80°C until RNA extractions were performed. The tumor cell percentage of the 38 pairs of breast cancer specimens are all >75%. None of the patients received chemotherapy or radiotherapy prior to surgery.

Total RNA was extracted according to the protocol of TRIzol reagent (Invitrogen Corporation, Beijing, China). DNA was extracted according to the protocol of AxyPrep™ Multisource Genomic DNA Miniprep Kit (Axygen Biosciences, CA, US). Quality and concentration of nucleic acid were measured by Gene Quant pro (GE Healthcare). cDNA was synthesized as described before [11].

### Real-time Quantitative PCR

Real-time quantitative PCR (qPCR) was performed using ABI 7000 sequence detection system (Applied Biosystems, Foster City, CA) in combination with the SYBR® Green PCR Master Mix (Roche, Indianapolis, USA), in accordance to the manufacturer’s recommendations. 1 µl cDNA or 10 ng genomic DNA from each sample was used as template in a PCR volume of 20 µl to detect ARID1A gene expression or copy number variation respectively. GAPDH and β-actin were applied as the internal reference for expression and copy number variation detection, respectively. Primers used for gene expression and copy number variation detection were listed in Table S2. Relative gene expression and copy number variation were calculated using the 2^−ΔΔCT^ formula. All experiments were performed in at least three biological replicates. Samples with a fold change 0.5 or less were defined as low expression or copy number loss, 2 or more were defined as high expression, 1.5 or more were defined as copy number gain (data can be found in Table S3). Methylated DNA immunoprecipitation was only detected in low or high expression samples.

### Methylated DNA Immunoprecipitation Assay

**Figure 1:**
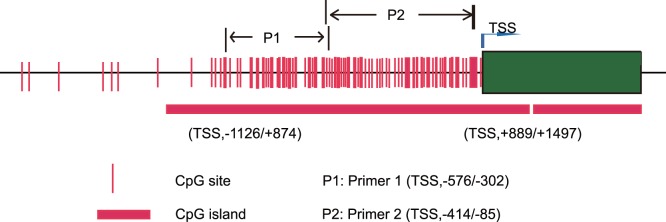
ARID1A promoter methylation. CpG site and island around transcription start site (TSS) were predicted by Methyl Primer Express. Two primer sets P1 and P2 were designed by Primer Primier 5 to amplify the TSS upstream CpG island.

ARID1A gene Promoter CpG islands were predicted with Methyl Primer Express (Applied Biosystems, CA, USA). Two pairs of primers were designed using Primer Primier 5 (PREMIER Biosoft, CA, USA) to amplify CpG islands of TSS (transcription start site) upstream (Figure 1). Methylated DNA immunoprecipitation (MeDIP) was carried out using EpiQuik Tissue Methylated DNA Immunoprecipitation Kit (Epigentek, Farmingdale, NY). We sonicated genomic DNA to produce random fragments ranging in size from 200 to 1000 bp. We used 1 µg of DNA for subsequent MeDIP enrichment. Briefly, DNA was denatured at 95°C for 10 min. Immunoprecipitation was then carried out at room temperature for 1 hour using 1 µg of monoclonal antibody against 5-methylcytosine (Epigentek, Farmingdale, NY) for the sample and 1 µg of normal mouse IgG as the negative control in a final volume of 100 µl antibody buffer. We incubated the mixture with 8-well assay strips supplied by the kit for 1 hour at room temperature and washed it twice with 150 µl of antibody buffer and wash buffer. We then treated the beads with proteinase K for 1 hour at 65°C and recovered the methylated DNA by phenol-chloroform extraction followed by ethanol precipitation. qPCR amplification was performed using Figure 1 described 2 primer sets (listed in Table S2). The assays were carried out in three replicates and relative methylation fold change was calculated for each sample with 2^−ΔΔCT^ formula. Detailed methylation data can be found in Table S4.

### Chromatin Immunoprecipitation Assay

Chromatin immunoprecipitation was performed using Chromatin Immunoprecipitation Kit (Millipore Corporation, Billerica, MA, USA,). In brief, Breast cancer tissue samples were cross-linked using 1% formaldehyde and terminated by the addition of 10× Glycine glycine. Homogenate and sonication were performed with Homogenizer (IKA, Staufen, Germany) and Ultrasonics Processors (Sonics, Newtown, USA). The chromatin was subjected to immunoprecipitation using the histone modification antibodies (Millipore): ChIPAb+ Trimethyl-Histone H3 Lys4 (H3K4Me3, Lot#NG1938454); ChIPAb+ Trimethyl-Histone H3 Lys27 (H3K27Me3, Lot#JBC1940033); ChIPAb+ Acetyl-Histone H3 Lys27 (H3K27Ac, Lot#1951080). Mouse IgG was used as a control. The magnetic beads were washed with wash buffers and TE buffer. DNA was finally eluted in elution buffer and used for real-time PCR amplification using the same primer sets with MeDIP-qPCR. The assays were carried out in three replicates and relative histone modification fold change was calculated for each sample with 2^−ΔΔCT^ formula. Detailed ChIP-qPCR data can be found in Table S5.

### PCR Product Sanger Sequence

The coding regions of ARID1A (CCDS285.1; NM_006015.4) were amplified by the PCR in 25 µl reactions containing 10× PCR Buffer, 2.5 mM dNTPs, 50 mM MgCl_2_, 10 µM forward and 10 µM reverse primers, 0.2 U Platinum® Taq (Invitrogen, San Diego, CA) and 10 ng DNA. The 28 pairs of primer sequences used were listed in Table S2. PCR cycling conditions were as follows: 94°C for 5 min; 45 cycles of 94°C for 30 s, 57°C for 30 s, 72°C for 1.5 min; followed by 72°C for 10 min.

PCR products were purified using Agarose Gel DNA Fragment Recovery Kit (TaKaRa Biotechnology, Dalian, China) and sequencing was carried out with Big Dye Terminator Kit v.3.1 (Applied Biosystems, Foster City, CA). One PCR primer of each pair was tagged with an M13F sequence (5′-GTAAAACGACGGCCAGT) to allow Sanger sequencing with this universal primer. Sequencing reactions were purified using the Agarose Gel DNA Fragment Recovery Kit (TaKaRa Biotechnology, Dalian, China) and run on ABI PRISM 3730 machines (Applied Biosystems, Foster City, CA). Variant Reporter™ Software (Applied Biosystems, CA, USA) was used to visually analyze sequencing traces for mutations. All mutation sites were conformed for second PCR and Sanger sequencing.

### Statistics

Based on expression data, we defined two groups: low expression group composed of all low expression samples, high expression group composed of all high expression samples. Methylation and histone modification levels, Copy number variation and mutation rate were all compared between these two groups. The statistical analysis was performed by using SPSS 11.5 software (SPSS Inc., Chicago, IL, USA). Data are expressed as means or numbers (percentages). Categorical variables were compared by Fisher exact test, and continuous variables were compared by Student *t* test. Two-tailed *P* values of less than 0.05 were considered statistically significant.

## Results

### ARID1A mRNA Low Expression was Common in the Invasive Ductal Carcinomas

ARID1A mRNA low expression (more than 2-fold decrease) was seen in 57.9% (22/38) invasive ductal carcinomas. This low expression group exhibited 2.7 fold higher Ki-67 protein expression by immunohistochemistry study compared with the high expression group (*p* = 0.011, in Table S6).

### ARID1A mRNA Low Expression was Strongly Associated with Promoter Hypermethylation of ARID1A Gene in Invasive Ductal Carcinomas

**Figure 2:**
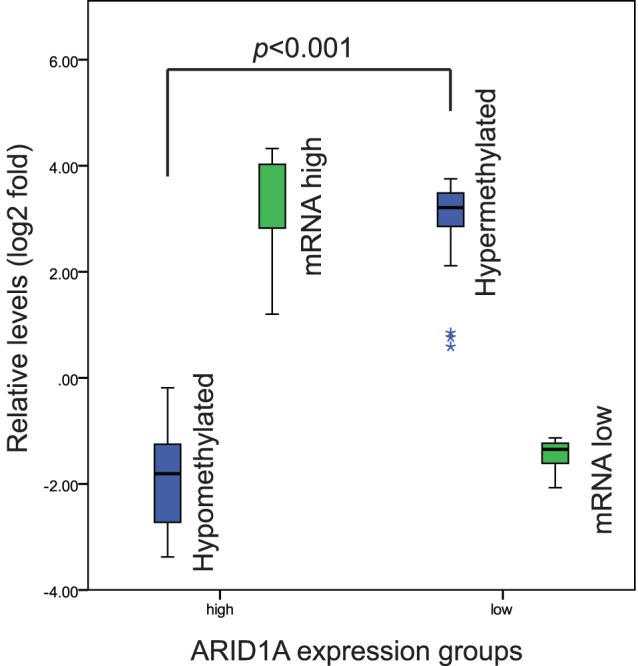
ARID1A gene promoter methylation levels between RNA high (n = 11) and low (n = 22) expression groups. Relative methylation levels were analyzed with MeDIP-qPCR. Promoter methylation levels (blue bar) were significantly higher in low expression group (green bar) compared with high expression group (green bar) (*p*<0.001).

Relative promoter methylation levels of ARID1A gene were evaluated with MeDIP-qPCR for ARID1A mRNA low or high expression groups. In low expression group, 86.4% (19/22) of patients exhibited promoter hypermethylation (more than 2-fold increase). In high expression group, 81.8% (9/11) of patients exhibited promoter hypomethylation (more than 2-fold decrease). The relative methylation fold changes were significantly different between low and high expression groups (*p*<0.001,student *t* test) (Figure 2).

### Repressive Histone Modification was Accompanied with Hypermethylation

**Figure 3:**
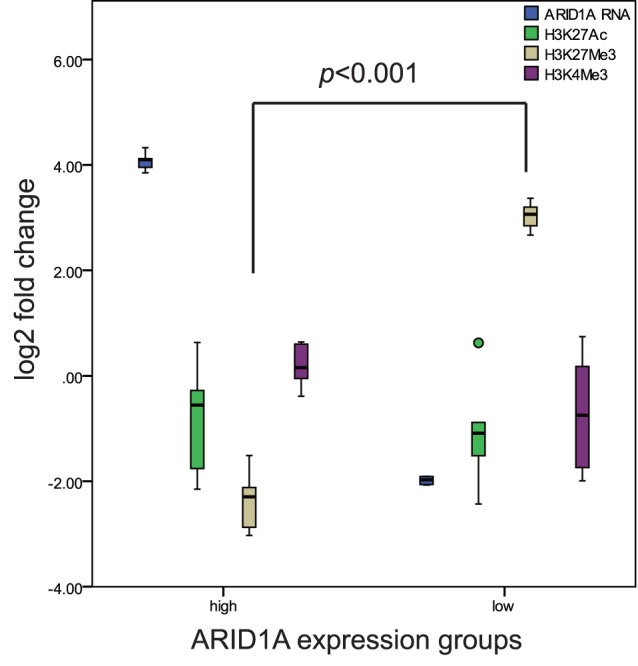
ARID1A gene promoter histone modification levels between RNA high (n = 5) and low (n = 5) expression groups. The histone modifications of H3K27Ac, H3K27Me3 and H3K4Me3 were analyzed with ChIP-qPCR. Only repressive marker H3K27Me3 was significantly higher (*p*<0.001) in low expression group compared with high expression group.

Histone modification is often accompanied with promoter methylation. In order to further support our findings in ARID1A promoter methylation, ChIP-qPCR was carried out to evaluate histone modification levels in the ARID1A top 5 low and top 5 high expressed samples respectively. Three types of histone modification were detected, including 2 types of repressive marker (H3K4Me3, H3K27Me3) and 1 type of active marker (H3K27Ac). The levels of H3K27Me3 was significantly higher in ARID1A mRNA low expression group compared with the high expression group (*p*<0.001,student *t* test) (Figure 3). Other differences in another two histone markers were not observed.

### ARID1A Gene Copy Number Variation was not Associated with ARID1A Gene mRNA Expression Levels

**Figure 4:**
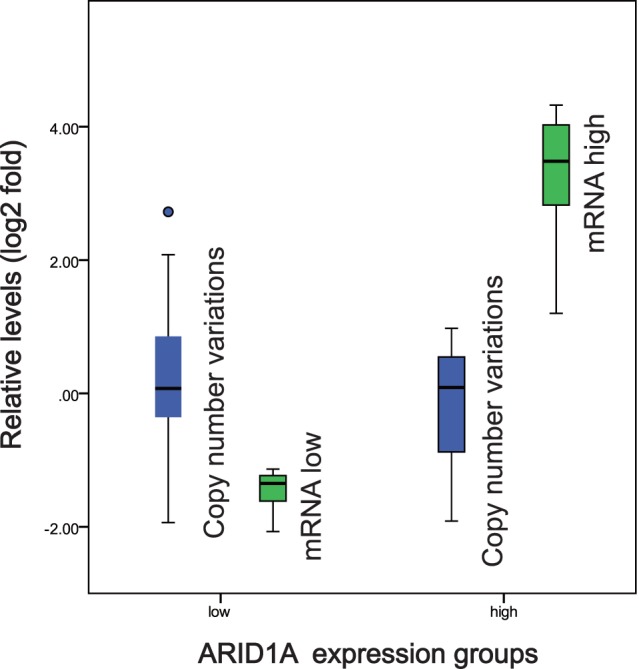
Copy number variations between ARID1A low and high expression groups. Samples were divided into low (n = 22) and high (n = 11) expression groups based on ARID1A mRNA relative expression (green). The difference of relative copy number variation (blue) was not significant between two groups (*p* = 0.274).

Copy number loss only occurred in five out of 38 (13.2%) invasive ductal carcinomas. Among them, one case was in ARID1A mRNA low expression group and three cases in the high expression group. Thus, there is no statistical difference between ARID1A mRNA low and high expression groups (Figure 4). This might be due to the limited numbers of cases showing the copy number loss. However, it cannot be excluded that copy number loss is not the cause for ARID1A gene low expression in invasive ductal carcinomas.

### Mutations in ARID1A Gene was not Associated with its mRNA Expression Levels

**Figure 5:**
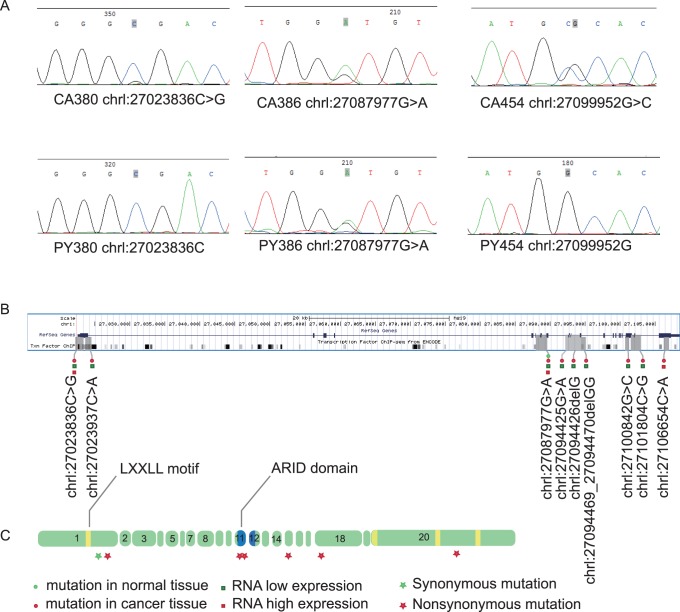
ARID1A mutation sites in genome and corresponding exon. (**A**)**.** Three mutation sites indentified with Sanger sequencing in three cancer (CA) and corresponding normal (PY) tissues. (**B**)**.** All the mutation sites indentified from our research were indicated in genome. Sample information, normal (green round spot) or cancer (red round spot) tissue, RNA low (green square) or high (red square) expression were also indicated. (**C**)**.** Mutation sites in exon. Protein synonymous (green star) and nonsynonymous (red star) mutations were indicated. Two of the mutation sits located in ARID domain.

With PCR product Sanger sequencing, we found 9 mutations in 10 of 33 pairs breast samples [Figure 5]. All mutation sites were not reported previously. Most of the mutation sites are somatic except the site chrl: 27087977G>A, which is germline mutation. 7 of 9 mutation sites locate in exon and another 2 locate in intron. Among the 7 exon mutation sites, 2 deletion mutation sites result in frameshift, 4 mutation sites result in amino acid change and 1 mutation site is synonymous mutation. Two frameshift mutation sites both locate in ARID domain. 24.2% (8/33) patients exhibited exon mutation. More detailed mutation site information can be found in Table S7. We didn’t see a significant mutation rate difference between low (31.8%, 7/22) and high expression (18.2%, 2/11) groups (*p* = 0.681, fisher exact test). But, the mutation rate obviously higher in low expression group.

## Discussion

ARID1A expression loss is common in many types of tumors [14]. Our previous study [11] indicates approximate 60% breast invasive ductal carcinomas exhibit ARID1A expression loss; this is in line with other report [12] and is associated with more aggressive phenotypes of breast cancer [12], alcohol intake of hepatocellular carcinoma [15] and chemoresistance of ovarian clear cell carcinoma [16]. Functional studies of ARID1A gene have proven that it’s a bona fide tumor suppressor gene both in breast cancer [12] and other types of cancers [10], [17].

Promoter hypermethylation is an important reason for tumor suppressor gene low expression in cancer [18], [19]. We showed a relatively higher promoter methylation level in low expression group compared with high expression group (*p*<0.001). 86.4% (19/22) of patients exhibited promoter hypermethylation in low expression group. Gene methylation is closely related with histone modification [20]. Our results indicated promoter hypermethylation was accompanied with repressive histone modification (H3K27Me3). This result further confirmed the presence of hypermethylation.

ARID1A gene haploinsufficiency has been reported in breast cancer recently [12], [13]. Our result is similar with one of the reports. But we didn’t see any difference in copy number variation between low and high expression group. 47% of pancreatic cancer tissues show ARID1A gene copy number loss, which is the main reason for low expression [10]. 58% of breast cancer tissues exhibited expression loss but only 13.2% of them exhibited copy number loss in this study. Copy number loss is not the main reason of ARID1A gene low expression yet in our current breast cancer research.

All the mutation sites detected in current study have not been reported previously [9], [13], [17]. We observed higher mutation rate in low expression group (31.8%) in contrast to high expression group (18.2%). But this difference trend has not statistical significance. Mutation is the main reason for low expression in colorectal [21] and ovarian cancer [8], [9]. But in breast cancer there is a big gap between exon mutation rate (24.2%) and expression loss rate (58%). So mutation is one reason for low expression but not the main reason.

One weakness of this study is that we didn’t perform mutation function research, especially mutations in ARID domain. Moreover, the sample size is not enough to detect real relations among haploinsufficiency, mutation and low expression. These all need to be studied in subsequent researches.

**Figure 6:**
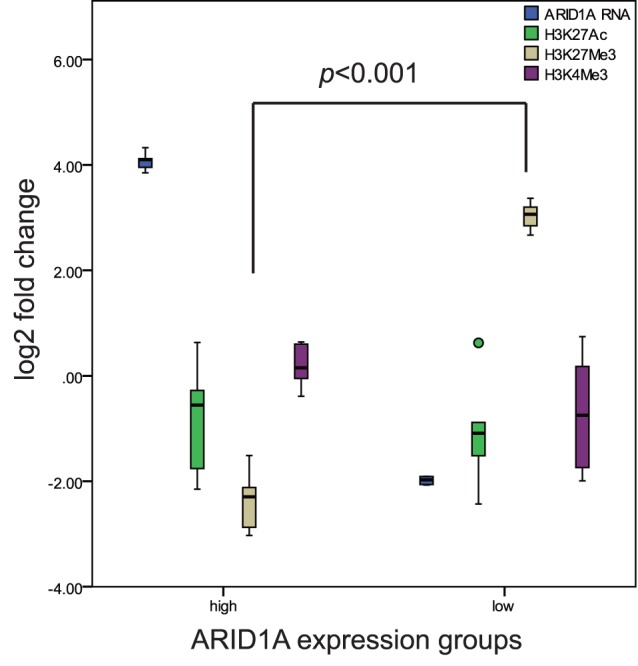
Different contributions to ARID1A mRNA abnormal expression. ARID1A relative mRNA, methylation, mutations and copy number variations were presented from outer to inner in doughnut chart. Percentage was indicated with length of circular arc.

In summary, we found promoter hypermethylation is the main reason for ARID1A gene expression loss in breast cancer (Figure 6). Mutation may also be an important reason. The contribution of different reasons to ARID1A low expression is different in different kind of tumor. Drug treatment chose and therapy effectiveness evaluation should all be based on the different contributions.

## Supporting Information

Table S1 **Clinical data of samples used in present study.**
(XLS)

Table S2 **Primers used for gene expression, copy number variation, Sanger sequencing, MeDip and CHIP qPCR.**
(XLS)

Table S3 **Real-time data of RNA expression and copy number variation.**
(XLS)

Table S4 **MeDip qPCR data.**
(XLS)

Table S5 **CHIP qPCR data.**
(XLS)

Table S6 **Clinical data comparison between ARID1A mRNA low and high expression group.**
(XLS)

Table S7 **ARID1A gene mutation sites indentified in breast cancer tissue.**
(XLS)
